# Anti-thyroglobulin antibody levels post-thyroidectomy and papillary thyroid carcinoma recurrence

**DOI:** 10.1186/s12885-025-14709-5

**Published:** 2025-08-25

**Authors:** Ho Jung Jeong, Jin Seok Lee, Jun Sung Lee, Hyeok Jun Yun, Hojin Chang, Seok Mo Kim, Yong Sang Lee, Hang-Seok Chang

**Affiliations:** 1 Department of Surgery, Ajou University School of Medicine, Suwon, Republic of Korea; 2 Department of Surgery, Gangnam Severance Hospital, Institute of Refractory Thyroid Cancer, Thyroid Cancer Center, Yonsei University College of Medicine, 211 Eonjuro, Gangnam-gu, Seoul, 135-720 Republic of Korea

**Keywords:** Anti-thyroglobulin antibody, Bilateral total thyroidectomy, Papillary thyroid cancer, Recurrence

## Abstract

**Background:**

The global incidence of thyroid cancer, particularly papillary thyroid carcinoma (PTC), is rising due to more frequent incidental findings. Despite a high 10-year survival rate of 93%, up to 28% of PTC patients experience locoregional recurrence. Postoperative monitoring typically relies on serum thyroglobulin (Tg), but the presence of anti-thyroglobulin antibodies (TgAb) interferes with Tg measurement, necessitating reliable detection methods. This study aimed to assess the predictive value of postoperative TgAb levels for PTC recurrence and establish a TgAb threshold as a prognostic marker.

**Method:**

A retrospective analysis was conducted on 15,620 patients who underwent bilateral total thyroidectomies at Gangnam Severance Hospital between March 2004 and December 2022. After excluding patients with unmeasurable postoperative TgAb or other carcinoma types, the final cohort comprised 4,434 PTC patients (775 men and 3,659 women, median age 46 ± 11.68 years). Bilateral total thyroidectomy was performed on all patients. TgAb levels were measured 2 days post-surgery and annually, with the most recent levels used for analysis. The primary outcome was PTC recurrence, analyzed based on stratified TgAb levels.

**Results:**

Elevated TgAb levels were significantly associated with increased tumor size and recurrence rates (*P* < 0.001). Patients with TgAb levels above 440 IU/mL showed a higher recurrence rate (13.3%) compared to those with lower levels. A TgAb threshold of 440 IU/mL was identified as a novel recurrence marker, with an odds ratio of 6.0 (95% CI: 2.987–12.053, *P* < 0.0001). The disease-free survival (DFS) was shorter in patients with TgAb levels above this threshold.

**Conclusions:**

Postoperative TgAb levels are a useful prognostic indicator for PTC recurrence, with a proposed threshold of 440 IU/mL serving as a predictive marker. This threshold provides valuable insight for monitoring patients, irrespective of test timing post-surgery, and may guide clinical decision-making for identifying high-risk patients.

**Supplementary Information:**

The online version contains supplementary material available at 10.1186/s12885-025-14709-5.

## Introduction

Thyroid cancer incidence is rising globally, primarily due to an increase in incidental findings of papillary thyroid carcinoma (PTC) [1]. PTC treatment has demonstrated excellent outcomes, with a 10-year survival rate of 93% [2]. Nevertheless, locoregional recurrences have been reported in up to 28% of patients despite a good long-term prognosis [3]. 

Because serum thyroglobulin (Tg) is exclusively produced in the thyroid tissue, it is a valuable biochemical marker in the postoperative surveillance of differentiated thyroid cancer (DTC) [4, 5]. Over the past few decades, anti-thyroglobulin antibodies (TgAbs) have garnered considerable attention. They have progressed from being a straightforward indicator of thyroid autoimmunity to essential for interpreting Tg levels, owing to their interference with its measurement [4–6]. A reliable detection of TgAbs is required before assessing the validity of a Tg measurement because the presence of TgAbs frequently results in artificially low blood Tg values. According to the most recent American Thyroid Association guidelines, an immunometric technique should be used to assess blood TgAb levels, in addition to serum Tg assays [7]. 

TgAbs have become more prevalent among patients with DTC in recent years, with a prevalence approximately twice as high as that in the general population [8, 9]. Some studies suggest individuals with thyroid nodules may be at higher risk of developing cancer if they have TgAbs [10, 11]. Compared with follicular thyroid cancer, PTC has a noticeably higher incidence of these antibodies [9]. Furthermore, the higher the TgAb level, the greater the chance of disease persistence or recurrence [12, 13]. The presence of TgAbs soon after thyroidectomy may also be linked to this risk. It should be noted that TgAbs may temporarily increase following surgery as an apparent immunological response to the procedure, and may also temporarily increase following radioiodine ablation therapy [14]. 

The amount of Tg secreted by thyroid tissue determines the concentration of TgAbs [15]. Monitoring TgAb levels can serve as a stand-in marker for DTC. The destruction of the thyroid tissue should eliminate the antigenic stimulation that promotes the development of TgAbs; therefore, their levels should be significantly reduced or even eliminated. According to recent research, patients whose TgAb concentration falls by more than 50% following surgery are at an extremely low risk for persistence or recurrence throughout the follow-up period. Furthermore, if the concentrations do not drop below 50%, the risk increases and may even increase if the TgAb level rises [16–20]. 

In this study, we analyzed the correlation between changes in TgAb levels after surgery and the PTC recurrence rate, and based on this, we suggest a threshold for TgAbs.

## Materials and methods

### Patients

**Fig. 1:**
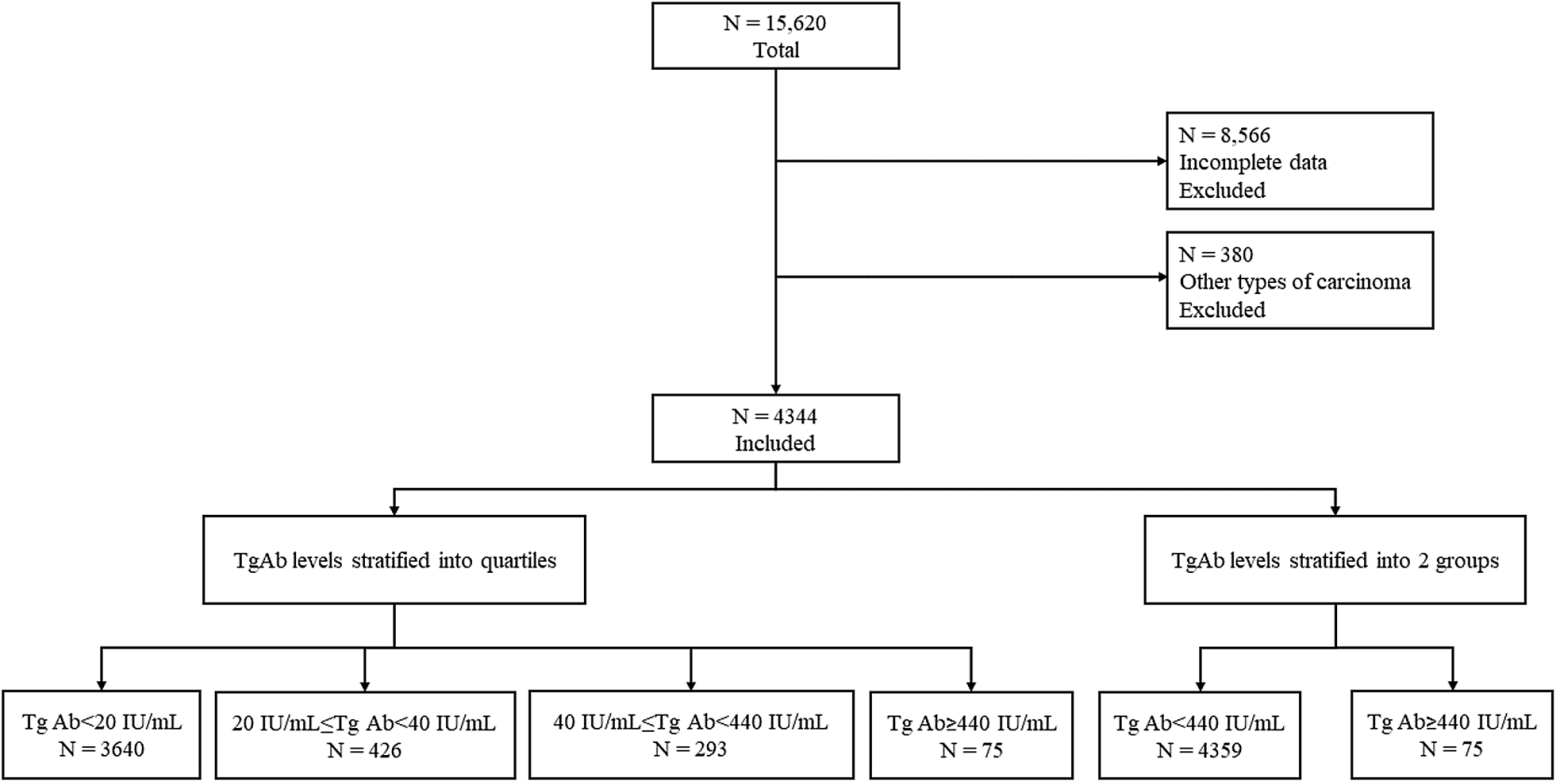
Flow chart showing the classification of 4434 patients into 4 groups and 2 groups according to TgAb level. The first group was divided into quartiles and the second group was divided into 2 groups

This retrospective study enrolled 15,620 patients with thyroid cancer who underwent bilateral total thyroidectomies for thyroid cancer at Gangnam Severance Hospital Thyroid Cancer Center between March 2004 and December 2022. Patients with unmeasurable postoperative TgAb levels due to missing data or undetectable assay limits (*n* = 8,566), as well as patients diagnosed with carcinoma types other than PTC (follicular carcinoma, medullary carcinoma, poorly differentiated carcinoma, anaplastic carcinoma, *n* = 380), were excluded. After exclusion, 4,434 PTC cases with a clear pathological diagnoses and complete medical records were retrospectively reviewed (Fig. 1).

The study procedures were approved by the Institutional Review Board (IRB) of Gangnam Severance Hospital, Yonsei University College of Medicine (approval number: 3-2024-0013). The study protocol was conducted in accordance with the Declaration of Helsinki. Due to the retrospective nature of the study, neither patient approval nor informed consent was required.

### Preoperative evaluation

Prior to surgery, all patients underwent thyroid ultrasonography to evaluate the size of the carcinoma and lymph node metastasis. Fine-needle aspiration biopsy was performed on suspicious nodules. Patients who were pathologically diagnosed with cancer and met the indications for surgery subsequently underwent surgical intervention.

### Laboratory tests and stratification

The first TgAb test was performed 2 days after surgery. Subsequent tests were conducted at intervals of approximately 1 year, and in this study, the last TgAb test for each patient was analyzed. Therefore, the follow-up periods for each patient were not consistent. The anti-thyroglobulin antibody levels were stratified into the following quartiles: ≤20 IU/mL, > 20 IU/mL to ≤ 40 IU/mL, > 40 IU/mL to ≤ 440 IU/mL, > 440 IU/mL. In these sections, the optimal cut-off point for predicting recurrence based on postoperative TgAb levels was established using concordance index (c-index) optimization. Several threshold values, including single and multiple points, were evaluated to identify the level that maximized the predictive accuracy for recurrence. The predictive performance of each threshold was quantified using odds ratios (OR) and the area under the receiver operating characteristic (ROC) curve (AUC) with corresponding 95% confidence intervals (CI). Among these thresholds, a TgAb level of 440 IU/mL demonstrated the highest c-index, with a statistically significant predictive capability for recurrence (OR = 6.0, 95% CI: 2.987–12.053, *p* < 0.0001), thus selected as the optimal threshold for subsequent analyses. (Table 1) (Fig. 1).

**Table 1:** Anti-thyroglobulin antibody (TgAb) cut-off point

Variable	Recurrence *N*/Total *N* (%)	OR (95% CI)	*P* value
TgAb			
< 20 IU/mL	91/3640 (2.5)	ref	
< 40 IU/mL	15/426 (2.52)	1.423 (0.817–2.481)	0.213
< 440 IU/mL	14/293 (4.78)	1.957 (1.1–3.48)	0.0223
≥ 440 IU/mL	10/75 (13.33)	6 (2.987–12.053)	< 0.0001

The same analysis as when dividing into four sections was performed using the TgAb level of 440 IU/mL, which showed the lowest *P*-value among these sections, as the standard.

### Statistical analysis

Data analysis was performed utilizing R software version 4.3.1 (R Project for Statistical Computing, Vienna, Austria). The TgAb level was regarded as a continuous variable, and its correlation with the odds ratio and probability of recurrence was illustrated through a scatter plot and spline curve. The relationship between TgAb levels and PTC recurrence was assessed using logistic regression analysis. The variables incorporated into the multivariate logistic regression model were chosen based on a combination of statistical significance derived from univariate analysis and clinical relevance. Variables exhibiting a *P*-value of less than 0.05 in univariate analysis, such as tumor size, central lymph node metastasis, lateral neck node metastasis, and serum Tg level were included. A clinically significant variable, radioiodine ablation (RAI) therapy, was also incorporated into the final model irrespective of its univariate significance. *P* < 0.05 was set as the threshold for statistical significance.

## Results

### Patient characteristics

Of the 4,434 patients, 775 were men and 3,659 were women, and the median age was 46 ± 11.68 years. Group 1 comprised 3,640 patients (TgAb level < 20 IU/mL), of which 2,957 (81.2%) were women and 683 (18.8%) were men. The median tumor size was 0.8 ± 0.72 cm, and 91 (2.5%) patients experienced cancer recurrence during the follow-up period. The proportion of women was higher than that of men in all other groups. In each group, increases in TgAb levels were accompanied by increases in tumor size, central lymph node metastasis rate, lateral neck lymph node metastasis rate, and cancer recurrence rate. However, the rate of extrathyroidal extension, RAI therapy and distant metastasis did not follow this trend (Table 2).

**Table 2:** Characteristics of patients of the study group divided into four groups(*n* = 4434)

		Tg Ab ≥ 440 IU/mL (*n* = 75)	40 IU/mL ≤ Tg Ab < 440 IU/mL (*n* = 293)	20 IU/mL ≤ Tg Ab < 40 IU/mL (*n* = 426)	Tg Ab < 20 IU/mL (*n* = 3640)	
Sex, n(%)	Female	70(93.3%)	262(89.4%)	370(86.9%)	2957(81.2%)	
	Male	5(6.7%))	31(10.6%)	56(13.1%)	683(18.8%)	
Age, median ± SD, years		41 ± 12.60	45 ± 12.26	46 ± 11.75	46 ± 11.59	
Operation type, n(%)	Conventional	74(98.7%)	291(99.3%)	426(100%)	3625(99.6%)	
	Endoscopic	0	0	0	2(0.1%)	
	Robot	1(1.3%)	2(0.7%)	0	13(0.3%)	
Tumor size, median ± SD, cm		1.3 ± 1.57	0.9 ± 1.14	0.8 ± 0.90	0.8 ± 0.72	
Extrathyroidal extension, n(%)		30(40.0%)	116(39.6%)	120(28.2%)	1168(32.1%)	
Central lymph node metastasis, n(%)		59(78.7%)	177(60.4%)	211(49.5%)	1659(45.6%)	
Lateral neck node metastasis, n(%)		34(45.3%)	72(24.6%)	88(20.7%)	564(15.5%)	
Radioiodine ablation therapy, n(%)		57(76%)	215(73.4%)	347(81.5%)	3281(90.1%)	
Follow up date, median ± SD, days		2071 ± 1432.74	1764 ± 1360.32	1738 ± 1101.63	2770 ± 1149.86	
Distant metastasis, n(%)		2(2.7%)	3(1.0%)	6(1.4%)	15(0.4%)	
Recur, n(%)		10(13.3%)	14(4.8%)	15(3.5%)	91(2.5%)	

When divided into two groups based on the cutoff value of 440 IU/mL, the group with a TgAb level below 440 IU/mL included 4,359 patients, and the group with a TgAb level above 440 IU/mL included 75 patients. In these two groups, there were 70 (93.3%) and 3,589 (82.3%) women, respectively; the proportion of women in the two groups was significantly higher than that of men (*P* = 0.001). In addition, tumor size (*P* < 0.001), central lymph node metastasis rate (*P* < 0.001), lateral lymph node metastasis rate (*P* < 0.001) and recurrence rate (*P* = 0.01) were all significantly larger or higher in the group with TgAb level above 440 IU/mL. Conversely, RAI therapy rate was higher in the group with TgAb level below 440 IU/mL (*P* = 0.002). Extrathyroidal extension (*P* = 0.192) and the rate of distant metastasis was higher in the group with TgAb level above 440 IU/mL, but this was not statistically significant (*P* = 0.106) (Table 3).

**Table 3:** Characteristics of patients of the study group(*n* = 4434)

		Tg Ab ≥ 440 IU/mL (*n* = 75)	Tg Ab < 440 IU/mL (*n* = 4359)	P value	
Sex, n(%)	Female	70(93.3%)	3589(82.3%)		
	Male	5(6.7%))	770(17.7%)	P = 0.001	
Age, median ± SD, years		41 ± 12.60	46 ± 11.66	P = 0.141	
Operation type, n(%)	Conventional	74(98.7%)	4342(99.6%)		
	Endoscopic	0	2(0.1%)		
	Robot	1(1.3%)	15(0.3%)	P = 0.265	
Tumor size, median ± SD, cm		1.3 ± 1.57	0.8 ± 0.78	P < 0.001	
Extrathyroidal extension, n(%)		30(40.0%)	1404(32.2%)	P = 0.192	
Central lymph node metastasis, n(%)		59(78.7%)	2047(47.0%)	P < 0.001	
Lateral neck node metastasis, n(%)		34(45.3%)	724(16.6%)	P < 0.001	
Radioiodine ablation therapy, n(%)		57(76%)	3843(88.2%)	P = 0.002	
Follow up date, median ± SD, days		2071 ± 1432.74	2668 ± 1200.39		
Distant metastasis, n(%)		2(2.7%)	24(0.6%)	P = 0.106	
Recur, n(%)		10(13.3%)	120(2.8%)	P = 0.01	

Table 4 presented the results of univariate and multivariate logistic regression analyses. In the univariate analysis, significant associations with recurrence were observed for tumor size (OR: 1.82; 95% CI: 1.58–2.09; *P* < 0.0001), central lymph node metastasis (OR: 1.14; 95% CI: 1.10–1.18; *P* < 0.0001), lateral neck node metastasis (OR: 3.63; 95% CI: 2.54–5.20; *P* < 0.0001), TgAb level between 40 IU/mL and 440 IU/mL (OR: 1.957; 95% CI: 1.10–3.48; *P* = 0.0223), and TgAb level ≥ 440 IU/mL (OR: 6.00; 95% CI: 2.987–12.053; *P* < 0.0001). RAI therapy did not show a statistically significant association with recurrence (OR: 1.50; 95% CI: 0.80–2.79; *P* = 0.206). In the multivariate analysis, tumor size (OR: 1.53; 95% CI: 1.30–1.80; *P* < 0.0001), central lymph node metastasis (OR: 1.10; 95% CI: 1.06–1.15; *P* < 0.0001), and TgAb level ≥ 440 IU/mL (OR: 4.13; 95% CI: 1.89–9.02; *P* = 0.0004) remained significant predictors of recurrence. Lateral neck node metastasis, RAI therapy, and lower TgAb level (< 440 IU/mL) were not statistically significant predictors in the multivariate analysis (Table 4).

**Table 4:** Univariate and multivariate logistic regression analysis of the study group

Variable	Univariable model			Multivariable model		
	OR(95% CI)	*P* value	AUC (95% CI)	OR(95% CI)	*P* value	AUC (95% CI)
Sex						0.78(0.74–0.82)
Female	ref					
Male	1.50(0.99–2.26)	0.054	0.533(0.496–0.57)			
Age	0.99(0.97-1.00)	0.1188	0.546(0.488–0.604)			
Tumor size	1.82(1.58–2.09)	< 0.0001	0.689(0.642–0.737)	1.53(1.30–1.80)	< 0.0001	
Central lymph node metastasis	1.14(1.10–1.18)	< 0.0001	0.718(0.671–0.765)	1.10(1.06–1.15)	< 0.0001	
Lateral neck node metastasis	3.63(2.54–5.20)	< 0.0001	0.626(0.583–0.669)	1.53(0.98–2.39)	0.0638	
Radioiodine ablation therapy	1.50(0.80–2.79)	0.206		1.24(0.66–2.33)	0.5120	
Tg Ab						
< 20 IU/mL	ref		0.568(0.526–0.61)	ref		
< 40 IU/mL	1.423(0.817–2.481)	0.213		1.19(0.65–2.18)	0.5804	
< 440 IU/mL	1.957(1.1–3.48)	0.0223		1.80(0.96–3.39)	0.068	
≥ 440 IU/mL	6(2.987–12.053)	< 0.0001		4.13(1.89–9.02)	0.0004	

In the study, after checking the Tg level in patients with TgAb levels below 40 IU/mL, the average Tg level was found to be 17.21 ng/mL, with a median ± SD of 0 ± 791.77 IU/mL. Central compartment neck lymph node dissection (CCND) was performed prophylactically for all patients. Additionally, RAI therapy was administered to almost all patients, and the doses showed minimal variation across the cohort.

### Correlation between postoperative TgAb level and PTC recurrence

**Fig. 2:**
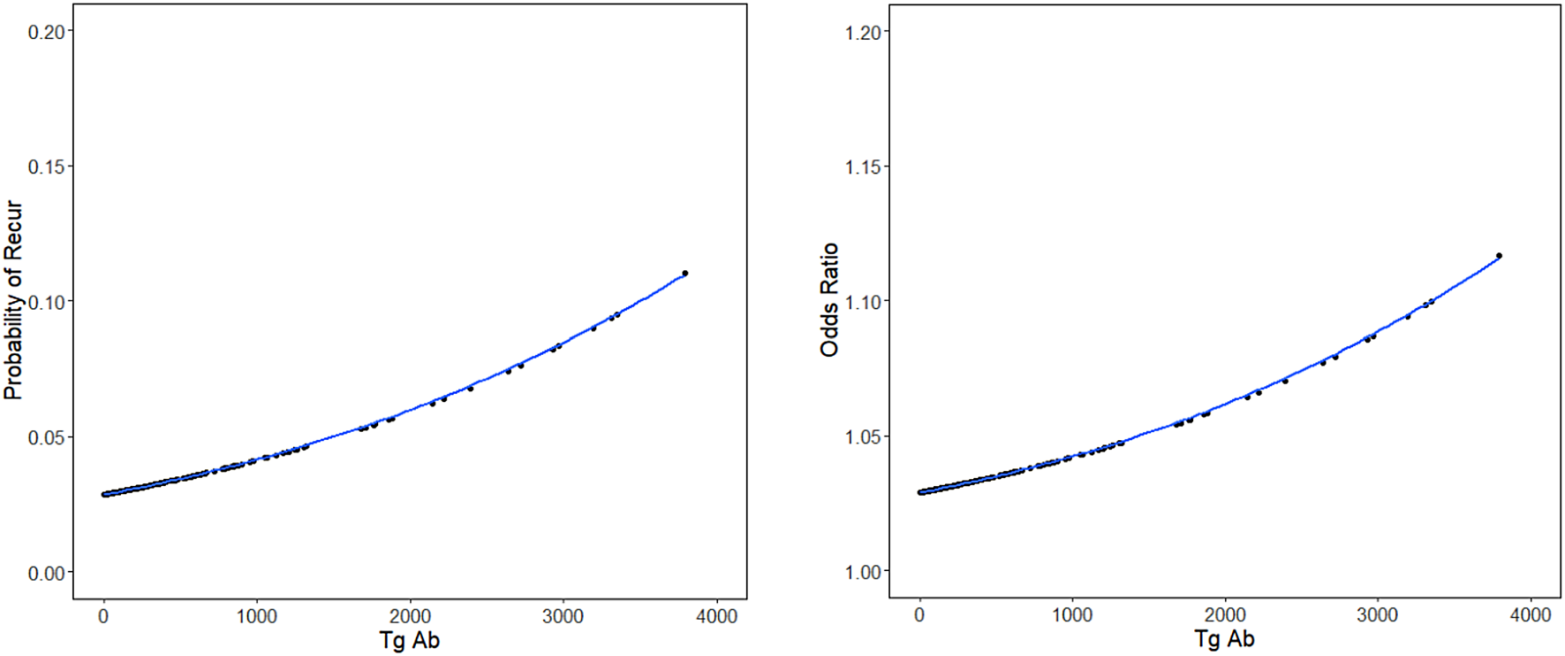
Spline curve and scatter plot showing the correlation between TgAb and odds ratio, probability of recur

The continuous nature of TgAb levels was examined, and the correlation between odds ratios and the probability of recurrence was depicted through a scatter plot and a spline curve (Fig. 2). The graphical representations reveal a consistent pattern: as TgAb levels increased, there was a corresponding elevation in the odds ratio and probability of recurrence.

### Survival analysis

**Fig. 3:**
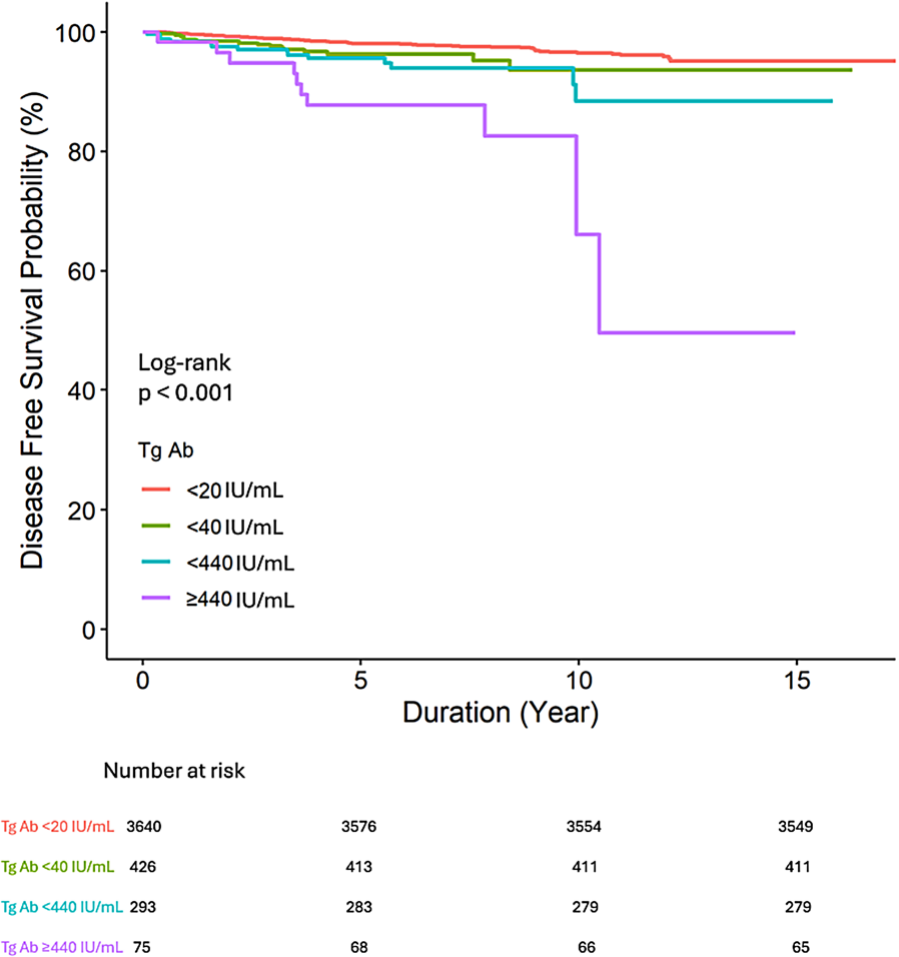
Disease-free survival of four groups patients

**Fig. 4:**
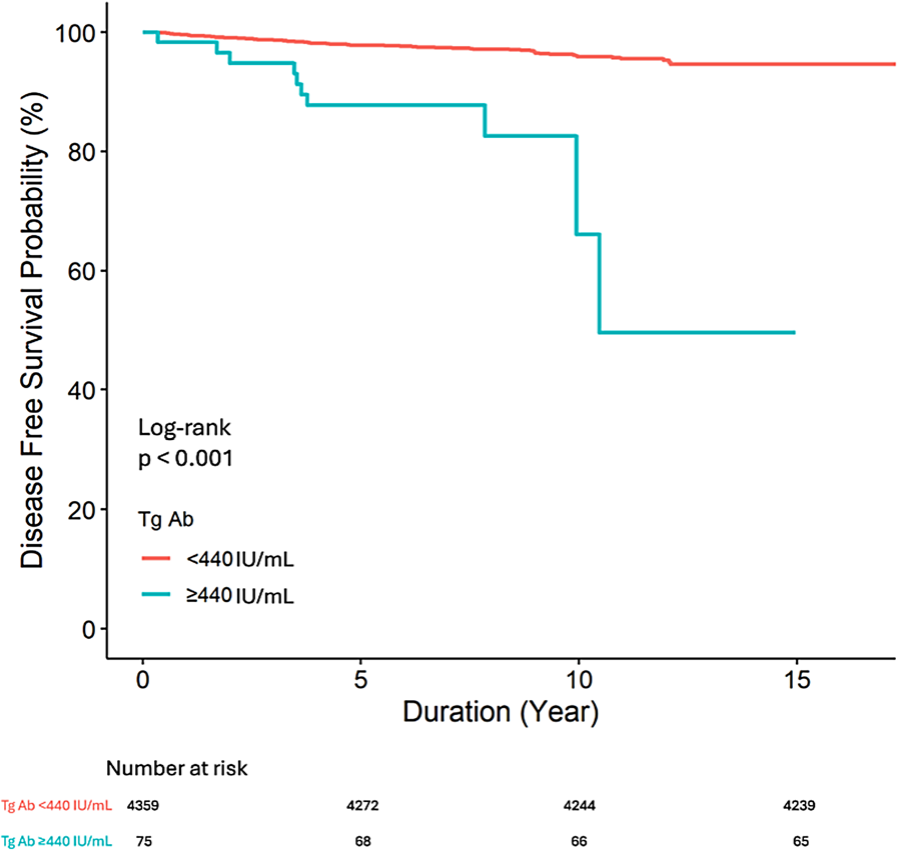
Disease-free survival of two groups patients

The disease-free survival (DFS) was analyzed using Kaplan-Meier survival curves (Fig. 3) of the groups divided into quartiles based on the TgAb levels demonstrated an inverse relationship: higher TgAb levels were associated with shorter disease-free survival periods. This trend aligned with the pattern observed in the scatter plots and spline curves in Fig. 2. Furthermore, the survival analysis performed following the categorization of patients into two distinct groups based on the cutoff value of 440 IU/mL revealed that the disease-free survival period of the group with TgAb level above 440 IU/mL was shorter than that of the group with TgAb level below 440 IU/mL (Fig. 4).

## Discussion

Previous reports of a higher incidence of elevated TgAb levels in patients with DTC suggest that thyroid tumor antigens are more prominently presented to the immune system; however, the exact cause is uncertain [21]. Following thyroidectomy and radioiodine ablation therapy, most patients with preexisting autoimmune thyroid disease lose their TgAb-positive status during follow-up, which is associated with removal of the thyroid tissue and its antigenic components. According to Gorges et al. [14] after 3 years, the percentage of patients with measurable TgAbs dropped to less than 10%, and the median serum half-life of TgAbs in patients with DTC receiving treatment was 10 weeks. Chiovato et al., [15] reported a median disappearance time of 3 years for TgAbs. Some studies have shown no significant association between serum TgAb levels and disease prevalence or progression, whereas others have proposed that continuously detectable TgAbs may indicate recurrence [12, 22–24]. Regarding the use of TgAbs as alternative tumor markers, there is no clear consensus [25, 26]. Managing patients with consistently increased TgAbs remains a clinical challenge.

Despite reports of variable TgAb prevalence in patients with DTC, no established threshold for positive TgAbs exists [27]. After ablation, Chung et al. [23] observed high TgAb levels in 22.6% of patients with DTC, with serum TgAb levels < 100 U/mL regarded as negative. However, Rubello et al. regarded TgAb levels < 50 U/mL as negative when they included 43 patients with DTC who had detectable circulating TgAbs at diagnosis prior to therapy [12]. After 6–12 months of treatment, 44.2% of the patients still had detectable TgAbs. A comparatively lower cutoff of 6 U/mL was employed by Gorges et al. as a positive result, and 29% of TgAbs were present during the first test [14]. Some authors have used a 1:100 titer as a positive TgAb criterion [15, 25]. A recent study by Aras et al. suggested an optimal threshold value of 27.8 IU/mL for TgAbs [28]. 

In the present study, we used a statistical program to set a cutoff value and conducted an analysis based on the collected data. We confirmed that higher TgAb levels corresponded to a higher the probability of PTC recurrence. Therefore, it is a good prognostic indicator in patients with PTC who have undergone total thyroidectomy. The main focus of this study was to suggest a new TgAb threshold that can help predict the recurrence of PTC after surgery.

Following total thyroidectomy, all follicular cells should be completely eliminated. This should stop the antigenic stimuli, which causes the concentration of TgAbs to decrease gradually until it is absent. Because all normal follicular cells have already been eliminated, TgAb persistence for an extended length of time following initial therapy or an increase in TgAb concentration suggests the persistence of Tg-producing tissues, which reflects persistent or recurrent DTC [16]. 

The therapeutic value of TgAbs in DTC is controversial. Some studies have employed less sensitive hemagglutination inhibition techniques [24, 25], whereas others have utilized different TgAb assays [23, 29]. According to recent guidelines, serum Tg should ideally be tested using the same assay in the same laboratory, and each time serum Tg is obtained, TgAb should be quantitatively evaluated [30]. According to many studies, there was no correlation between low TgAb levels and poor postoperative outcomes. TgAbs were evaluated immediately after surgery or resection, and patients with metastatic disease were not excluded [8, 9, 14, 15]. The transition to a TgAb-negative state may be take time, even in the absence of residual thyroid tissue. Examining variations in TgAb levels may be complicated by ongoing antigenic stimulation at recognized metastatic sites. To allow for a natural drop in TgAb levels and use these levels as a predictive indicator in patients without a theoretically feasible source of Tg, this study exclusively included patients who had undergone complete thyroidectomy.

This study has some limitations. First, we did not measure preoperative TgAb levels in all patients. The preoperative TgAb level would have been a more reliable control value, compared with the TgAb concentration at the time of remnant ablation. This is because the TgAb concentration is likely to be unstable owing to the opposing influences of decreased thyroid mass and acutely increased Tg during surgery. In this context, further studies comparing preoperative TgAb levels and TgAb values in the early postoperative period are warranted. Second, most tumors were small. However, central and lateral lymph node metastasis were observed in 47.5% and 17.1% of patients, respectively, indicating that the study population included a range of disease severity. Third, this is a retrospective study.

In conclusion, serum TgAb levels may serve as a useful predictor of recurrence in patients who have undergone total thyroidectomy for papillary thyroid cancer. Based on the observed pattern that higher serum TgAb levels correspond to higher recurrence rates, this value alone can be used as a new prognostic indicator, regardless of the timing of the TgAb test after surgery. The optimal threshold point for TgAb as a novel marker was determined to be 440 IU/mL.

## Supplementary Information

Below is the link to the electronic supplementary material.

Supplementary Material 1

## Data Availability

Data is provided within the supplementary information files.Authors are willing to make their data, analytical methods, and study materials available to other researchers on reasonable requests.

## Abbreviations

PTC: Papillary thyroid carcinoma
Tg: Thyroglobulin
TgAb: Anti-thyroglobulin antibodies
DTC: Differentiated thyroid cancer
OR: Odds ratios
ROC: Receiver operating characteristic
AUC: Area under receiver operating characteristic curve
CI: Confidence intervals
DFS: Disease-free survival
CCND: Central compartment neck lymph node dissection
RAI: Radioiodine ablation
